# Odontogenic Myxoma of the Maxilla: A Report of Unusual Pediatric Case

**DOI:** 10.5005/jp-journals-10005-1123

**Published:** 2011-04-15

**Authors:** Vijeev Vasudevan, Usha Mohan Das, V Manjunath, Radhika Manoj Bavle, M Sudhakar, Nanda Kumar, Srinath Srinath

**Affiliations:** 1 Professor and Head, Department of Oral Medicine and Radiodiagnosis, Krishnadevaraya College of Dental Sciences and Hospital, Bengaluru, Karnataka, India; 2 Former Dean, VS Dental College and Hospital, Bengaluru, Karnataka, India; 3 Senior Lecturer, Department of Oral Medicine and Radiodiagnosis, Krishnadevaraya College of Dental Sciences and Hospital, Bengaluru, Karnataka, India; 4 Professor and Head, Department of Oral Pathology and Microbiology, Krishnadevaraya College of Dental Sciences and Hospital, Bengaluru, Karnataka, India; 5 Senior Lecturer, Department of Oral Pathology and Microbiology, Krishnadevaraya College of Dental Sciences and Hospital, Bengaluru, Karnataka, India; 6 Principal, Professor and Head, Department of Oral and Maxillofacial Surgery, Krishnadevaraya College of Dental Sciences and Hospital, Bengaluru, Karnataka, India; 7 Professor, Department of Oral and Maxillofacial Surgery, Krishnadevaraya College of Dental Sciences and Hospital Bengaluru, Karnataka, India

**Keywords:** Maxillary sinus, Multilocular, Odontogenic myxoma, Odontogenic tumor.

## Abstract

Odontogenic myxoma (OM) is a rare and locally benign neoplasm of high aggressive behavior found exclusively in the jaws. OM commonly occurs in the second and third decade, its quite rare to find in maxilla that to invading the maxillary sinus completely. The lesion often grows without symptoms and presents as a painless swelling. The radiographic features are variable, and the diagnosis is therefore not easy. This article presents a case of OM of maxilla in a 13-year-old boy, which was previously diagnosed as fibrosseous lesion with the help of CT.

## INTRODUCTION

Myxoma is a benign tumor of primitive mesenchymal tissue, closely mimicking the structure of mucoid connective tissue of umbilical cord. The odontogenic myxoma is a rare, benign tumor which does not show metastasis but local agressiveness and involves the maxilla and mandible. When involving the maxilla, odontogenic myxomas can invade the maxillary sinus, and are then diagnosed later stages only after having grown to huge mass.^1^ According to literature review, odontogenic myxomas (OM) represent between 1% and 17.7% of all odontogenic tumors.^2^

## CASE REPORT

**Fig. 1:**
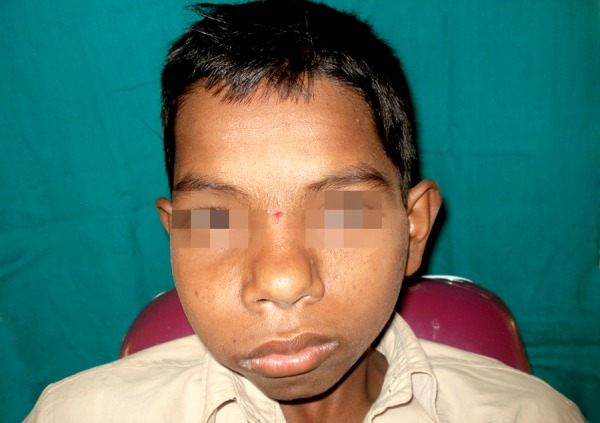
Painless swelling on the right maxilla

**Fig. 2:**
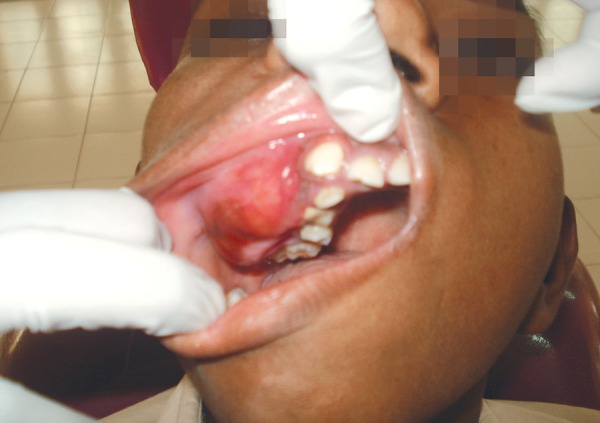
Bony hard swelling extending from the maxillary right lateral incisor to maxillary tuberosity

A 13-year-old male was referred to the Dental Department at Krishna Devaraya College of Dental Health Sciences Center for definitive management of a right-sided maxillary lesion. Which was previously diagnosed as fibrosseous lesion with the help of CT 2 years back. A three-year history of a slow growing mass causing intermittent pain in the right midface was reported. The patient denied any visual disturbance. Physical examination revealed fullness of the right midface which was mildly tender to palpation. The overlying skin was not erythematous and he demonstrated no lymphadenopathy or trismus (Fig. 1). Intraoral examination revealed a firm, nontender swelling expanding the buccal cortex of the maxilla, extending from right lateral incisor to second molar, there was no mobility in the overlying teeth, but displacement of teeth was noted, measuring around 4 x 2 cm in diameter (Fig. 2).

**Fig. 3:**
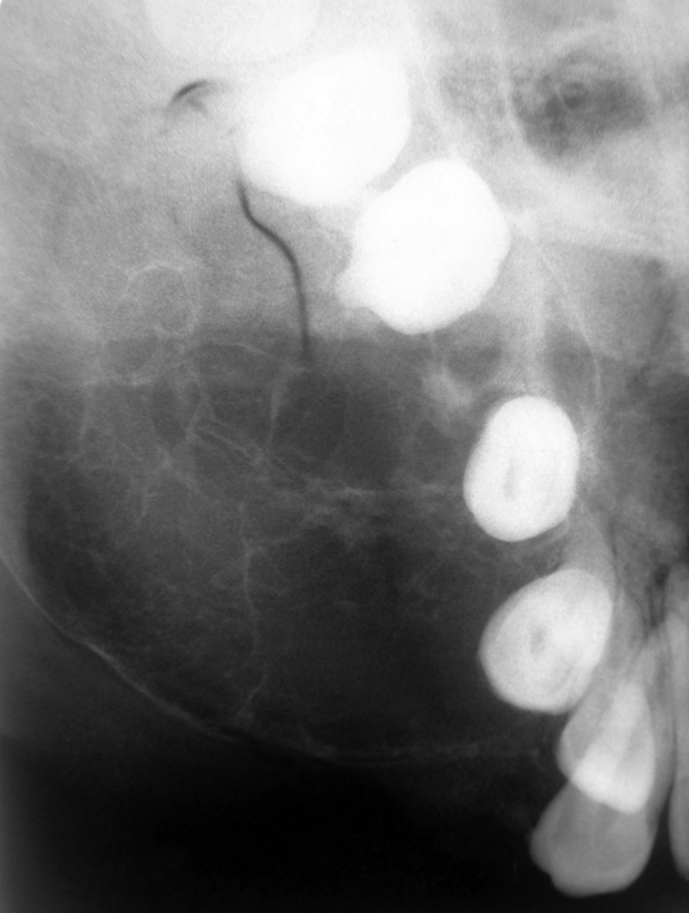
Occlusal radiograph showing a multilocular radiolucent lesion with tennis racket pattern

The occlusal radiograph showed a large multilocular radiolucent area with a well-defined sclerotic margin extending from the right lateral incisor to the distal aspect of the right second molar, with ’spider web’ and ‘tennis racket’ pattern appearance, with which a preliminary diagnosis of OM was made (Fig. 3). A computed tomographic (CT) scan, axial and coronal views demonstrated an lytic lesion with expansion and thinning of the overlying buccal cortex with radiopaque foci spread throughout the lesion involving the right maxillary antrum (Fig. 4).

**Fig. 4A:**
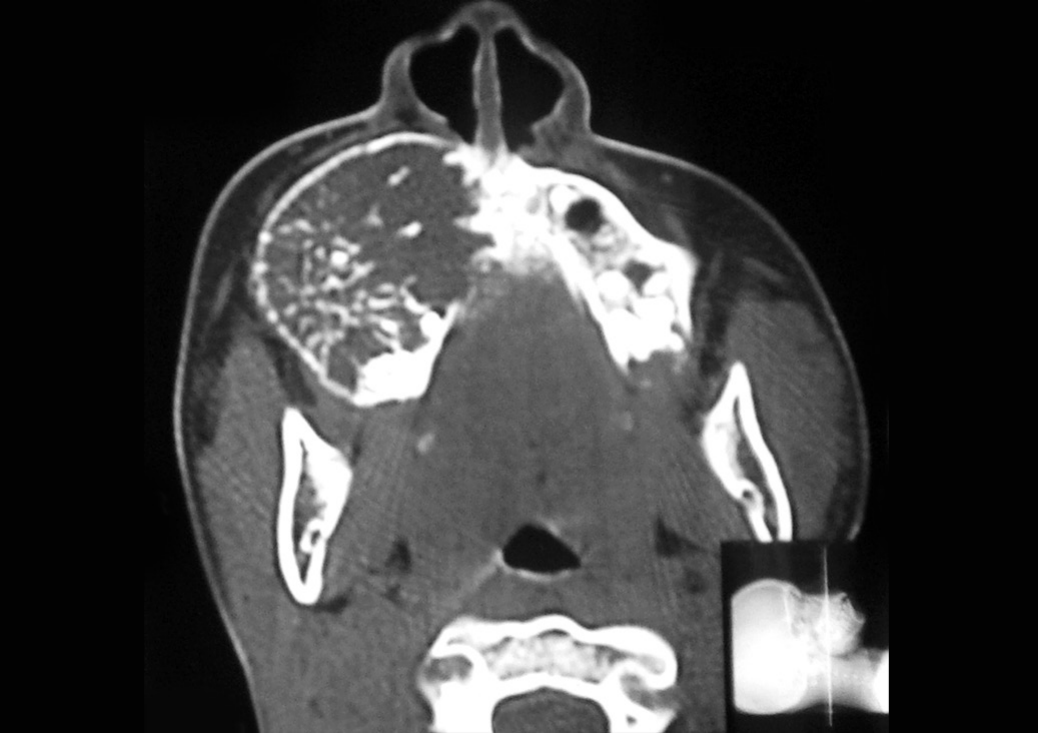
Coronal and axial views showing the lesion with obliteration of right maxillary sinus

**Fig. 4B:**
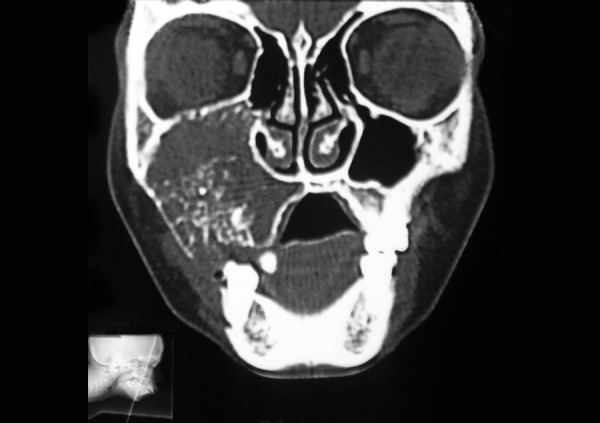
Coronal and axial views showing the lesion with obliteration of right maxillary sinus

**Fig. 5:**
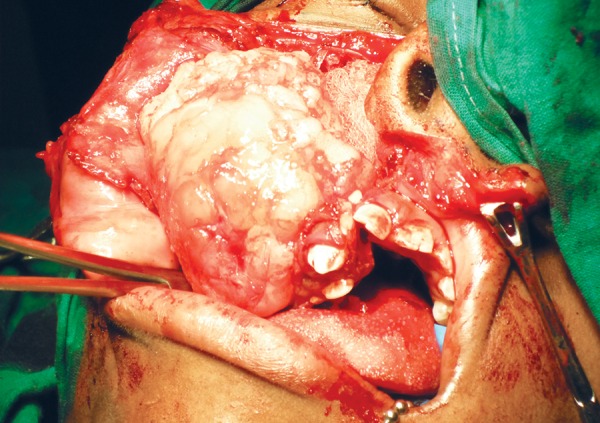
Intraoperative view showing tumor mass

**Fig. 6:**
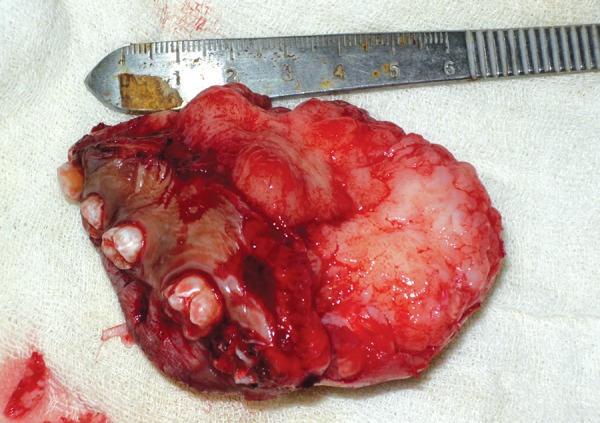
Gross-resected specimen of the gelatinous mass with glistening mucoid substance

**Fig. 7:**
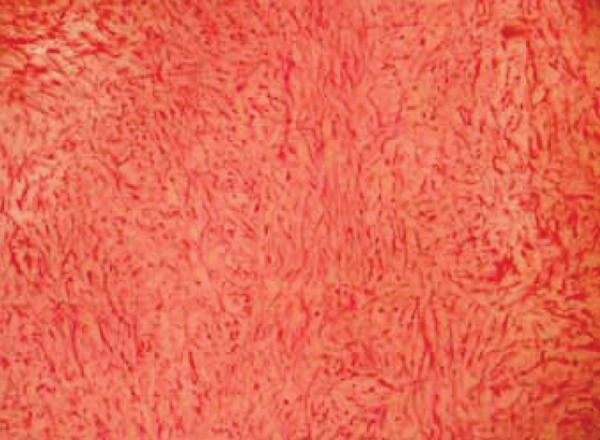
HE section at high power magnification showing a loose myxoid stroma embedded with haphazardly arranged stellate Spindle-shaped and round cells

An incisional biopsy confirmed the diagnosis of odontogenic myxoma. The surgical management involved a combined intra- and extraoral approach. The tumor was resected with a margin of normal tissue. This involved a maxillary ostectomy (Fig. 5). Macroscopically, the surgical specimen consisted of a segment of complete right maxilla and antrum with gelatinous mass with glistening mucoid substance (Fig. 6). Microscopically, the tumor was composed of loosely arranged spindle cells with serpentine nuclei within a variably myxoid and fibrous stroma (Fig. 7). Postoperative recovery was uneventful. The patient has since been seen regularly for follow-up, and treatment planning for dental rehabilitation is currently underway. He will be monitored long-term for signs of recurrence clinically and radiographically.

## DISCUSSION

Myxoma is a benign, mesenchymal-stemed, slowly prolif-erative, local aggressiveness and high rate of recurrence. Virchow coined this term in 1863, which was subsequently defined by him as “Schleimgesch-wulste” (myxomata) in 1871, because he thought of only soft tissue myxoma, as it happens with the umbilical cord, and had mucin. Myxomas may involve both hard and soft tissues.^3,4^ Myxoma is defined as a true neoplasm composed of rounded, angular, stellate and sometimes spindle-shaped cells set in a myxoid stroma containing mucopolysaccharide through which course very delicate reticulin fibers in various directions.^5^ According to the WHO’s classification of odontogenic tumors, in 1992, the Myxoma is considered a tumor of the odontogenic mesenchyme with or without the presence of odontogenic epithelium.^6^ Usually, when it involves bony tissue, it affects the facial bones.^7^ Odontogenic myxoma is believed to originate from the dental papilla or follicular mesenchyme. The evidence for its odontogenic origin arises from its location in the tooth bearing areas of the jaws, its occasional association with missing or unerupted teeth and the presence of odontogenic epithelium.^8^

Odontogenic maxillary myxoma was first mentioned in the literature by Thoma and Goldman in 1947. OM usually affect adolescents and young adults, between the second and third decades of life, very rarely affects people below 10 years of age or above 50 years. With equal gender predilection, mandibular tooth bearing area is favored over the maxilla.^9,10^ The present case reported here can be of unusual variant because, the patient’s history of 3 years duration of lesion, which might have started in early age and site of lesion also, i.e. maxillary posterior segment with involvement of antrum. OM clinically present as an asymptomatic lesion discovered during routine dental or radiological examinations in the initial stages of lesion or as a lesion associated with painless jaw expansion in later stages. Lesions with more advanced stages may be associated with pain, paresthesia, facial asymmetry, ulceration, teeth displacement and root resorption^11,12^ our case showed only facial asymmetry and displacement of teeth. So, the clinical differential diagnosis should include ameloblas-toma, odontogenic keratocyst, radicular cyst, dentigerous cyst lateral periodontal cyst, intraosseous hemangioma, simple bone cyst, giant cell granuloma, aneurismal bone cyst and metastasis of malignant tumors which shows the slow growth pattern.^13^

Macroscopically, the lesion appears to be a soft gelatinous yellowish grey mass which is often nonencapsulated. Cut surface of the lesion exhibits characteristic slimy appearance. Histopathologically, OM consists of triangular stellate cells with long processes intermeshing with each other. The intercellular matrix is mucoid, and the cytoplasm is slightly basophilic, finely granular and with a well-defined nucleus, mitotic figures are few. Cells may show pleomorphism. Bone may be rarely present with islands of inactive odontogenic epithelium.^14^ Recent ultrastructural studies have showed that myxoma is a tumor of fibroblasts, modified in such a way to form a matrix composed of glycosaminoglycans and do not form collagen fibrils-designated as ‘myxoblasts’. Histological differential diagnosis should be made with rhabdomyosarcoma, myxoid liposarcoma, neurogenic sarcoma, neurofibroma, lipoma, fibroma, chondromyxoid and nodular faciitis.^15,10^ Conventional radiography (oc-clusal and orthopantomography) and CT are useful in the detection of OM and also helpful to estimate the size, extent and margins of the tumor. Because of wide variation of radiographic presentation of OM, apart from most specific radiographic patterns, CT must be used to confirm conventional radiographic findings. Literature studies have described this tumor as both a unilocular or multilocular radiolucency and as having a distinct or diffuse margin.^16^ Barros in 1969, proposed two stages of radiologic patterns, first stage osteoporotic appearance, with more prominent medullary spaces separated by thin septa of bone. During this stage, the lesion acquires its classic radiographic appearance, consisting of multilocular radiolucency with well-developed locules, composed of trabeculae tending to intersect at right angles, forming locules straight, thin, elongated and lacy, Eversole called this as ‘Lichen planus of jaw bone’. It has varied radiographic presentations: Soap-bubble or honeycomb, spider web, tennis racket appearances. Other shapes include small or large triangles, diamonds, squares, rectangles, and X,Y and V figures. Second stage consists of the breakout or destructive phase consisting of loss of internal locules, significant expansion and perforation of the cortex with invasion into surrounding soft tissues. in maxilla there is extension into the antrum. Sometimes the peripheral margin of the septa may be arranged at right angles to the margin, giving a ‘hair brush’ or ’sun burst’ appearance.^17^ Due to its varied radiographic feature, the present case was misdiagnosed as fibroseous (fibrous dysplasia) lesion based on CT findings which led to delay in accurate treatment leading to vast anatomic destruction, leading to facial disfigurement. OM should be considered in the differential diagnosis of both radiolucent and mixed radiolucent-radiopaque lesions of both jaws in all age groups.^1^ Radiographic differential diagnosis of odontogenic Myxoma should include: Intraosseous heman-gioma, cherubism, aneurismal bony cyst, fibrous dysplasia, ameloblastoma, central giant cells lesion, traumatic bony cyst and odontogenic cysts (radicular, lateral periodontal, dentigerous and keratocyst).^18^ The current treatment for OM includes resection with bony margins of at least 1.0 to 1.5 cm and leaving behind one uninvolved anatomic boundary. Maxillectomy and sometimes resection of the orbital floor are required for OM in the upper jaw.^19^ Treatment for the present case was same. Enucleation, curettage and peripheral ostectomy are inadequate because of its gelatinous and nonencapsulated nature which makes the lesion to recur. Period of the greatest recurrence rate is seen in the first 2 years.^20^ Defects resulting from maxillary resection can be replaced by prosthetic obturator or tissue reconstruction procedures.^21^

Due to varied radiographic presentation it makes difficult to diagnose lesion based on radiographic features alone. Proper diagnosis requires clinical, histological and radiographic correlation. Because of its high rate of recurrence, especially due to its gelatinous, nonencapsulated and mucous aspect, surgical treatment through bone resection is the most indicated treatment modality, and the patient must be followed up closely for years.
